# Community-engaged artificial intelligence: an upstream, participatory design, development, testing, validation, use and monitoring framework for artificial intelligence and machine learning models in the Alaska Tribal Health System

**DOI:** 10.3389/frai.2025.1568886

**Published:** 2025-04-07

**Authors:** Brian Travis Rice, Stacy Rasmus, Robert Onders, Timothy Thomas, Gretchen Day, Jeremy Wood, Carla Britton, Tina Hernandez-Boussard, Vanessa Hiratsuka

**Affiliations:** ^1^Department of Emergency Medicine, Stanford University, Palo Alto, CA, United States; ^2^Center for Alaska Native Health Research, University of Alaska Fairbanks, Fairbanks, AK, United States; ^3^Maniilaq Association, Kotzebue, AK, United States; ^4^Alaska Native Tribal Health Consortium, Anchorage, AK, United States; ^5^Department of Medicine, Stanford University, Palo Alto, CA, United States; ^6^Southcentral Foundation, Anchorage, AK, United States

**Keywords:** American Indian and Alaska Native, community engaged research, artificial intelligence, ethical considerations in AI, medevacs, emergency care, rural health, mixed methods

## Abstract

American Indian and Alaska Native (AI/AN) communities are at a critical juncture in health research, where combining participatory methods with advancements in artificial intelligence and machine learning (AI/ML) can promote equity. Community-based participatory research methods which emerged to help Alaska Native communities navigate the complicated legacy of historical research abuses provide a framework to allow emerging AI/ML technologies to align with their unique world views, community strengths, and healthcare goals. A consortium of researchers (including Alaska Native Tribal Health Consortium, the Center for Alaska Native Health Research at University of Alaska, Fairbanks, Stanford University, Southcentral Foundation, and Maniilaq Association) is using community-engaged AI/ML methods to address air medical ambulance (medevac) utilization in rural communities within the Alaska Tribal Health System (ATHS). This mixed-methods convergent triangulation study uses qualitative and quantitative analyses to develop AI/ML models tailored to community needs, provider concerns, and cultural contexts. Early successes have led to a second funded project to expand community perspectives, pilot models, and address issues of governance and ethics. Using the Ethical, Legal, and Social Implications of Research framework to address implementation of AI/ML in AI/AN communities, this second grant expands community engagement, technical capacity, and creates a body within the ATHS able to provide recommendations about AI/ML security, privacy, governance and policy. These two projects have the potential to provide equitable AI/ML implementation in Alaska Native healthcare and provide a roadmap for researchers and policy makers looking to effect similar change in other AI/AN and marginalized communities.

## Introduction

Indigenous communities, and Alaska Native communities in particular, are at an exciting crossroads in health research. Participatory methods are converging with rapidly advancing technological in artificial intelligence and machine learning (AI/ML) and creating opportunities to advance equity. The equity benefits of AI/ML use for Alaska Native healthcare can be achieved through participatory research and implementation practices that account for accessibility in the human resources, connectivity, software and hardware needed within Alaska Native healthcare settings with upstream inquiry and attention in design and implementation to social and data biases (Prathomwong and Singsuriya, 2022).

## Health research in Alaska Native communities

Alaska Native people come from a long history of strength and resilience, where communities have overcome unique challenges for millennia to thrive in their historical lands (Rasmus et al., 2019; Williams, 2009). The remoteness and resilience of Alaska Native peoples made them a subject of medical study, but early medical research in Alaska was a troubling mix of violations of trust, lack of informed consent, and active harms (Foulks, 1989; Institute of Medicine and National Research Council, 1996; Lanzarotta, 2020). These past injustices created a pathway and precedence for extractive research relationships where outside researchers enter Alaska Native communities and collect data to address a problem or study a phenomenon at the researchers direction (Gaudry, 2011; Crouch et al., 2023).

Alaska Native and American Indian (AI/AN) federally recognized Tribes have implemented research policies reflecting their community values and requirements in the conduct of research with their citizens and in their lands and healthcare facilities (Carroll et al., 2022; Garba et al., 2023; Saunkeah et al., 2021). Self-governing tribes are implementing processes and systems to support Indigenous data sovereignty, meaning the right of each Native nation to govern the collection, ownership, and application of the Tribe’s data (Carroll et al., 2019). Community-based participatory research (CBPR) methods have concurrently evolved as a response to inequitable power structures in research with communities by using cyclical processes to build upon community strengths, facilitate partnerships, promote co-learning, and look at health from positive and ecological perspectives (Satcher, 2005). CBPR methods have revolutionized health research being done by Alaska Native communities looking at a wide range of health domains and shaping solutions to problems most highly prioritized among Alaska Native people living in the communities (LaVeaux and Christopher, 2009; Rasmus, 2014; Woodbury et al., 2019).

## AI/ML in healthcare

Globally, a technological revolution is occurring surrounding AI/ML applications in healthcare which have the potential to transform the efficiency and precision of care delivery and improve health outcomes (Bohr and Memarzadeh, 2020). However, equity concerns exist for AI/ML in regards to minority health and health disparities. Historically marginalized populations have been underrepresented in the datasets used in the training of AI/ML models and in the inclusion of end users providing healthcare to marginalized populations, which has negatively impacted real-world implementation and impeded health outcomes improvements (Obermeyer et al., 2019; Sharma et al., 2022). AI/ML designers who create models which do not consider biases present in datasets and the social structures from which the data are derived (e.g., inequalities due to and at the intersections of race, class, gender, sexuality, disability, and coloniality) can reinforce or worsen existing health disparities (Green et al., 2024; Donia and Shaw, 2021).

However, an emerging body of scholarship is exploring ways in which AI/ML can be used to actively address and reduce health disparities (Chen et al., 2020; d’Elia et al., 2022; Berdahl et al., 2023). AI/ML development to address health disparities should adhere to several key principles: (a) be built from equitable datasets that represent marginalized groups, (b) considerations of equity must occur at all stages of algorithm development, (c) development teams need to be more diverse in terms of stakeholders and backgrounds, and (d) ethical standards must be established (Green et al., 2024). While frameworks and a few participatory cases in AI/ML exist, the practical implementation of AI/ML in AI/AN healthcare systems is almost entirely missing from the literature (Birhane et al., 2024). A scoping review showed that only 0.2% of over 1,000 AI healthcare publications mention community involvement, and only a single study has been published describing stakeholder involvement in application development (Loftus et al., 2024). To date, no studies or frameworks have been published with community engagement throughout the design and implementation process, nor any involving AI/AN health systems.

## Practical implementation of AI/ML in rural AI/AN healthcare

A consortium of academic and community-based researchers is now looking at how to leverage the successes of CBPR within Alaska Native communities to build equitable AI/ML models within the Alaska Tribal Health System (ATHS) that could benefit Alaska Native patients, be rooted in Alaska Native community beliefs, recognize the unique local healthcare environment, and be built using Alaska Native health data. This team includes researchers from the Alaska Native Tribal Health Consortium, the Center for Alaska Native Health Research at University of Alaska, Fairbanks, Stanford University and two Tribal health organizations (THO): Southcentral Foundation, and Maniilaq Association. Our research consortium has received funding from the National Institute of Minority Health and Health Disparities to attempt to use CBPR approaches in a mixed-methods convergent triangulation design to study air medical ambulance (or “medevac”) utilization in rural Alaska. Over 80% of Alaska’s communities are in frontier locations, unconnected to a road system. Without roads, medevacs are required to transfer medical emergencies from rural village-based health clinics with healthcare delivered solely by Community Health Aides and Community Health Practitioners (CHA/Ps) to a referral hospital to receive a higher level of care (Sherry, 2004; Chernoff and Cueva, 2017; Trout et al., 2018). With medevacs representing a critically limited resource which carry not just high financial costs but also substantial crash risks, the question of how and when to medevac a patient is the most challenging clinical task facing clinicians in rural Alaska (Rice et al., 2020).

Medevac utilization provides an ideal pilot for “community-engaged AI” as it is: (a) a clear priority for patients and providers within the ATHS, (b) an issue unique to rural Alaska and thus requiring the use of data from rural Alaska, (c) an extraordinarily complex problem involving weighing many clinical (e.g., current and potential patient conditions, vital signs, patient complaints, provider exam) and non-clinical (e.g., provider training, resource availability, weather, aircraft availability) determinants, and (d) a health service that is Tribally managed.

**Figure 1:**
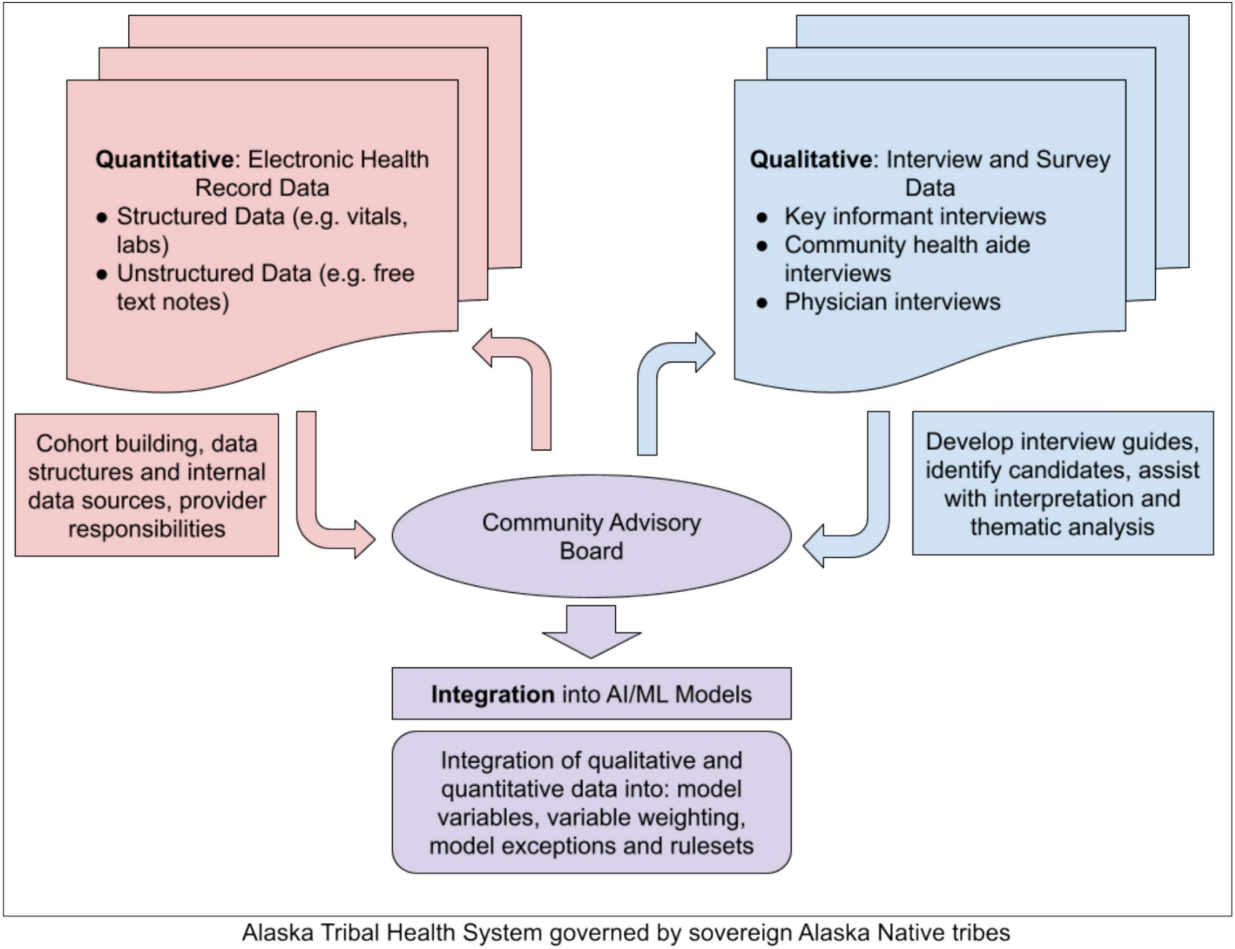
Concurrent triangulation mixed-methods design.

AI/ML models represent a technical solution to deciphering this complex decision-making process. While AI/ML models are notorious for requiring a large amount of data, these needs can be adequately met despite the relatively small population in rural Alaska by using the comprehensive health records encompassing a patient’s lifespan and inclusion of medical, dental, and behavioral health services, that the ATHS has maintained for decades (Carroll et al., 2011). Furthermore, the ATHS is a closed system where final outcomes for every patient can be accounted for, greatly improving the quality of the overall data. Quantitative analysis will connect medevac patients to their final outcomes and attempt to isolate which archetypes of patients have time-sensitive conditions which most benefit from medevac prioritization (Rice et al., 2020). Qualitative analysis of interviews with hospital-based physicians and the CHA/Ps who staff remote village health clinics will establish how and why both provider groups make their medevac decisions. This information will allow researchers to identify and weigh variables to include in their final models. A community advisory board will provide guidance and assist researchers with interpretation. Importantly, our project occurs within the structural socio-political setting of AI/AN communities where Alaska Native people operate and govern the ATHS. Our community-engaged AI/ML project is grounded by Alaska Native values. Beyond the community advisory board, key personnel of our research team work in research departments based at THOs and are AI/AN community members and health researchers. The proposed concurrent triangulation mixed-methods design is visualized in Figure 1.

The integrative stage will incorporate the qualitative and quantitative analyses to establish to build a final AI/ML classification model for medevac appropriateness. By associating the output of this model with patient-level mortality and mortality outcomes, the impact of medevac utilization on patient outcomes can be determined. The features of this model that are associated with better or worse patient outcomes can be incorporated into ongoing training. This model in turn will be the basis for further work to provide clinical decision support for this complex challenge faced by patients and providers throughout rural Alaska.

## Expansion of AI/ML implementation efforts in AI/AN communities

**Figure 2:**
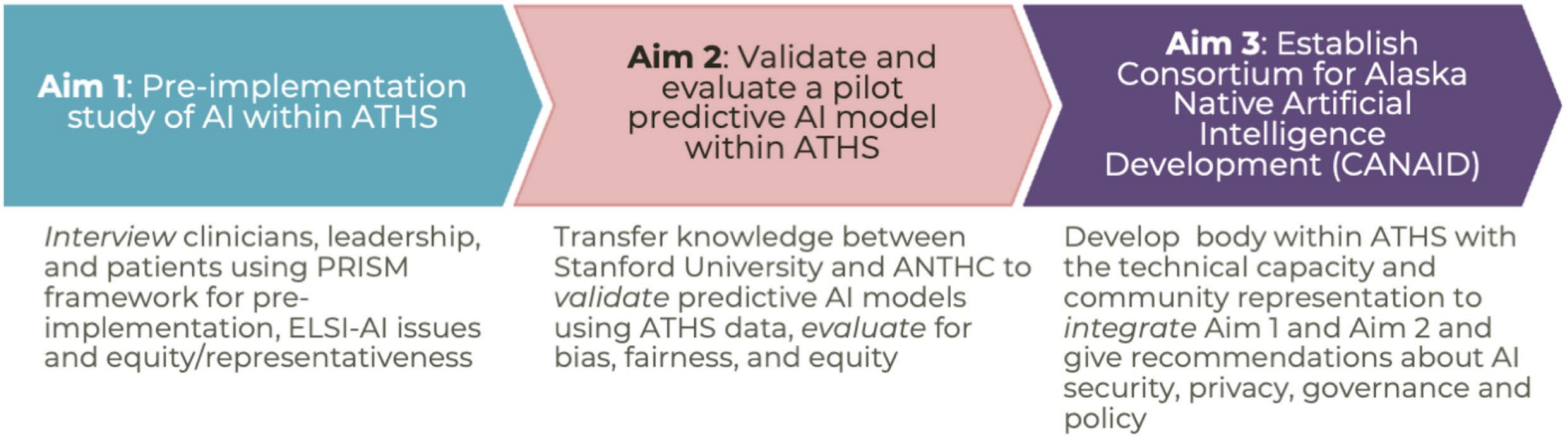
AIM-AHEAD project.

Though this work is only in its third year, the initial positive response from Alaska Native communities and leadership has led to expanded participation of additional THOs, and the pilot data has led to follow-up funding from the Artificial Intelligence/Machine Learning Consortium to Advance Health Equity and Researcher Diversity Program (AIM-AHEAD) (AIM-AHEAD, 2024). This second grant builds off preliminary data from the original project and will expand both the scope of community engagement, distribution of power, and expand technical capacity within community partnerships, as well as begin addressing system-wide issues of Tribal data sovereignty, policy and governance (Figure 2).

This work uses an upstream, embedded Ethical, Legal and Social Implications Research (ELSI) AI/ML framework to facilitate stakeholder engagement and use of empiric ELSI findings in all aspects of the research project to support collaboration, partnership, trust building, accessibility, ownership, accountability and transparency (Rajagopalan et al., 2024; Wiens et al., 2019). The work will expand the qualitative methods of the initial project to include the community not just of providers, but of the larger patient population. It also provides formal pre-implementation evaluation using frameworks for AI, helps pilot predictive AI/ML models within the ATHS, and establish broader guidance for ethics and governance for AI/ML projects. The research team will take externally developed predictive AI models for patient outcomes in cardiovascular disease and cancer and assess their performance, fairness, and bias when using ATHS data. The team will either develop mitigation strategies to adapt these models for use within the ATHS or establish the need for building and training AI models for the ATHS within the ATHS, using ATHS data. While AI/ML research often lacks clear clinical applicability, these projects are notable for not only being developed with support from patients, providers, researchers, and local and statewide Tribal leadership, but also with clear pathways for subsequent implementation within the ATHS to improve training for providers and provide direct clinical benefits to Alaska Native patients.

## Discussion

The methods described above have applicability beyond medevacs and beyond Alaska. It is hoped that publishing this work in its preliminary stages will provide a starting point for other under-represented and minority communities interested in engaging in AI/ML research to address the health issues that are unique to their communities. CBPR-driven AI/ML research may avoid *a priori* assumptions in design and locally situated models that are respectful and reflective of Alaska Native people and values. The process of co-design will allow the research team and community to explore specific design decisions, describe and mitigate real AI/ML harms, determine specific ATHS systemic inequalities, and support transparency and communication of AI/ML with end users. Embedding ELSI AI/ML into the project may expand the field in regard to normative research with historically marginalized communities using AI/ML in healthcare (Parker et al., 2019). Ultimately, the aim is to create an accessible and equitable pathway for Alaska Native people to gain the benefits promised by AI/ML and eliminate existing health disparities. It is hoped that this work can help provide a roadmap for other communities to do the same.

## Data Availability

The original contributions presented in the study are included in the article/supplementary material, further inquiries can be directed to the corresponding author.
